# Comparative Analysis of Whole Chloroplast Genomes of Three Common Species of *Echinochloa* (Gramineae) in Paddy Fields

**DOI:** 10.3390/ijms232213864

**Published:** 2022-11-10

**Authors:** Yuan Gao, Guohui Shen, Guohui Yuan, Zhihui Tian

**Affiliations:** Eco-Environmental Protection Research Institute, Shanghai Academy of Agricultural Sciences, Shanghai 201403, China

**Keywords:** *Echinochloa*, chloroplast genome, single-nucleotide polymorphisms, repeat, phylogenetic tree

## Abstract

*Echinochloa crus-galli* var. *crus-galli*, *E. crus-galli* var. *zelayensis*, and *E. glabrescens*, morphologically similar at the seedling stage, are the most pernicious barnyard grass species in paddy fields worldwide. Chloroplast (cp) genomes could be conducive to their identification. In this study, we assembled the complete cp genome sequences of *Echinochloa crus-galli* var. *crus-galli* (139,856 bp), *E. crus-galli* var. *zelayensis* (139,874 bp), and *E. glabrescens* (139,874 bp), which exhibited a typical circular tetramerous structure, large and small single-copy regions, and a pair of inverted repeats. In *Echinochloa crus-galli* var. *crus-galli,* there were 136 simple sequence (SSRs) and 62 long (LRs) repeats, and in the other two species, 139 SSRs and 68 LRs. Each cp genome contains 92 protein-encoding genes. In *Echinochloa crus-galli* var. *crus-galli* and *E. glabrescens,* 321 and 1 single-nucleotide polymorphisms were detected compared to *Echinochloa crus-galli* var. *zelayensis*. IR expansion and contraction revealed small differences between the three species. The phylogenetic tree based on cp genomes demonstrated the phylogenetic relationship between ten barnyard grass species and other common Gramineae plants, showing new genetic relationships of the genus *Echinochloa*. This study provides valuable information on cp genomes, useful for identifying and classifying the genus *Echinochloa* and studying its phylogenetic relationships and evolution.

## 1. Introduction

The genus *Echinochloa* Beauv (barnyard grass) is an annual or perennial gramineous plant widely distributed worldwide. This genus contains approximately 50 of the most pernicious weed species in global crops, especially in rice (*Oryza sativa*) fields [1,2,3,4], where they are very successful competitors, mainly because their ecological evolution is similar to that of rice [5,6]. The rice yield reduction caused by the wanton occurrence of barnyard grass may be very serious, making it the most troublesome weed for rice farmers [7,8]. *Echinochloa crus-galli* (L.) P. Beauv, *E. crus-galli* var. *zelayensis* (Kunth) Hitchc., and *E. glabrescens* Munro ex Hook. f. are the most common weeds in the middle and lower reaches of the Yangtze River, China, one of which often forms a dominant species in paddy fields. These three barnyard grass species are very similar in morphology at the seedling stage and can hardly be identified unless they are in heading stage. However, there are differences in the sensitivity to herbicides among different barnyard grass species [9,10], and the seedling stage of barnyard grass is the key management period. Therefore, it is important to identify the type of barnyard grass at the seedling stage.

Presently, the classification of the genus *Echinochloa* is mainly based on external forms such as spikes, small ears, awn length, and seed morphology [11,12]. However, external forms cannot distinguish many types of barnyard grass [13]. Several scholars believe that studying chromosomes and biochemical data helps identify barnyard grass species because the chromosome number of different barnyard grass species is not similar, and the number of chromosomes of some barnyard grass species is 2*n* = 54, while others are 2*n* = 36 [14,15,16]. With the rapid development of molecular biology techniques, new techniques have been applied to identify barnyard grass species. Inter-simple sequence repeat (ISSR) has also been widely used for genetic diversity analysis and germplasm identification [17,18]. Lu et al. performed molecular identification of 53 samples of barnyard grass species collected from 14 provinces and regions in China using ISSR technology and showed that the taxa of barnyard grass in rice areas have a certain genetic basis [19]. Yamaguchi et al. used the non-coding gene sequence trnt-l-f to investigate the molecular system of barnyard grass species in East Asia and divided the nine barnyard grass species into five groups [13]. In summary, this genus has no unified and widely recognized classification standard. Wu et al. conducted an in-depth analysis of the genetic evolution of barnyard grass as a weed and an orphan crop through genomics, showing its complex role in evolution [20]. In addition, owing to different agricultural farming methods, crop growth characteristics, geographical location, and herbicide use, barnyard grass from different regions also have many differences in seed germination rate, flowering time, leaf area, plant height, spikelet length, aboveground biomass, root weight, and seed quantity [21,22], which further complicates the classification of barnyard grass.

Chloroplasts (cps), organelles in photosynthetic plants or algae, contain genetic material, and their genomes are highly conserved because of haploidy, uniparental inheritance, and no recombination, providing abundant evolutionary information [23,24,25,26]. In addition, the cp genome is small and easy to obtain completely compared to the nuclear genome; therefore, its research is worthy in species identification, population genetics, and phylogeny [27]. Due to these common characteristics, identifying and analyzing ribosomal tissues in chloroplast systems has become an important method for solving plant phylogeny and evaluating biodiversity [28,29,30]. The cp genome has a typical quadripartite structure, large single-copy (LSC) and small single-copy (SSC) regions separated by the region of inverted repeats (IRs), which are a pair of sequences with opposite orientations, named IRa and IRb [31,32,33,34,35]. Sequences between the IRa and IRb regions can generate triggered flip-flop recombination, stabilizing single-copy regions [36]. Studies have shown that plant cp genomes are particularly helpful in characterizing the phylogeny and history of most plant lineages in reticular evolution (hybridization) and polyploidy [37,38,39]. With the development of chloroplast genome sequencing technology and an in-depth understanding of chloroplast genomes by researchers, the genetic relationships of many plant species have been clarified, such as the genera *Camellia*, *Taxodium*, and *Pterocarpus* [27,36,40]. To date, information regarding the phylogenetic relationship and evolutionary direction of the genus *Echinochloa* based on chloroplasts has been limited.

In this study, the cp genomes of *E. crus-galli* var. *zelayensis* and *E. glabrescens* were sequenced for the first time. This is also the first complete analysis and comparison of the cp genomes of three common *Echinochloa* weeds, *E. crus-galli* var. *crus-galli*, *E. crus-galli* var. *zelayensis,* and *E. glabrescens*, collected in paddy fields, which provides a convenient method for the identification of these three morphologically consistent plants and is also beneficial in identifying the choice of herbicides for individual weeds. Simultaneously, this study also conducted a systematic development analysis of the cp genome of many *Echinochloa* species in the NCBI database, which provides a theoretical basis for the regeneration of diversity and resource utilization of the genus *Echinochloa*.

## 2. Results

### 2.1. Differences in the Phenotype of Seeds

**Figure 1:**
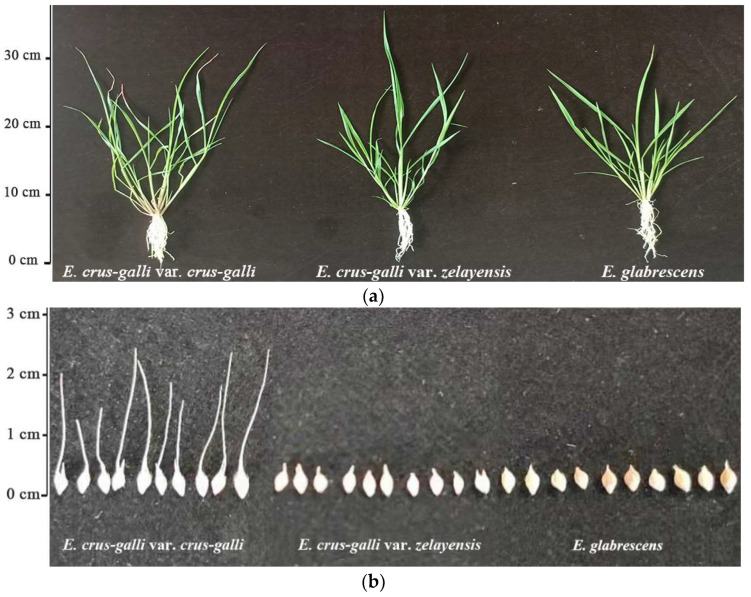
Morphology of three barnyard grass species. (**a**). Comparison of morphology of shoots. (**b**). Differences in the morphology of seeds.

In this study, the awn length and dry weight per 1000 grains of the seeds of *E. crus-galli* var. *crus-galli*, *E. crus-galli* var. *zelayensis,* and *E. glabrescens* were determined (Figure 1). The awn length of the *E. crus-galli* var. *crus-galli*, *E. crus-galli* var. *zelayensis*, and *E. glabrescens* were 1.63, 0.00, and 0.00 cm, respectively (Table 1). The dry weight per 1000 seeds of *E. crus-galli* var. *crus-galli*, *E. crus-galli* var. *zelayensis*, and *E. glabrescens* were 2.806, 2.139, and 3.006 g, respectively (Table 1). The awn length of *E. crus-galli* var. *crus-galli* was significantly longer than those of the other two species (*p* < 0.05). In addition, the dry weight per 1000 seeds of *E. glabrescens* was significantly higher than those of the other two species (*p* < 0.05).

### 2.2. Differences in Sensitivity to New Herbicides

**Figure 2:**
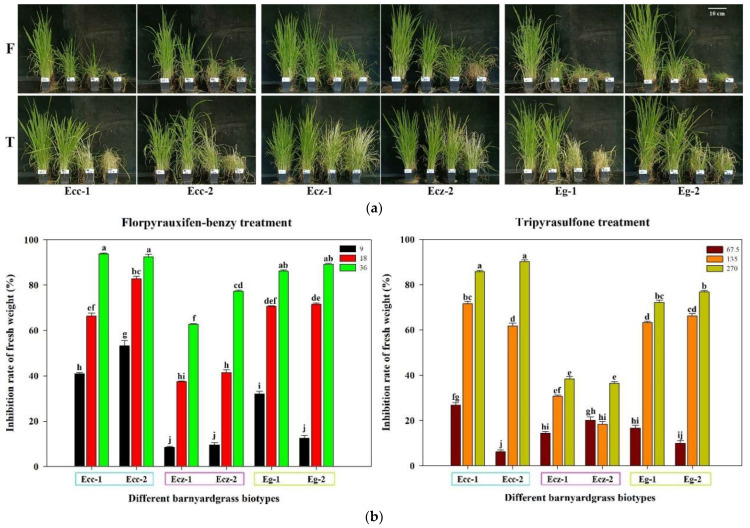
Differences in sensitivity to florpyrauxifen-benzyl and tripyrasulfone among *E. crus-galli* var. *crus-galli*, *E. crus-galli* var. *zelayensis*, and *E. glabrescens*. “F” means florpyrauxifen-benzyl treatment and the doses from left to right are 0, 9, 18, and 36 g a.i. ha^−1^. “T” means tripyrasulfone treatment and the doses from left to right are 0, 67.5, 135, and 270 g a.i. ha^−1^. “Ecc” means *E. crus-galli* var. *crus-galli.* “Ecz” means *E. crus-galli* var. *zelayensis.* “Eg” means *E. glabrescens*. ”-1” means biotype 1. ”-2” means biotype 2. ANOVA significance groupings were shown as a–j. (**a**) Differences in morphology after florpyrauxifen-benzyl and tripyrasulfone treatment. (**b**) Fresh weights of plants from each biotype at the end of equivalent treatment periods plotted as a percentage of the respective control.

The sensitivity of three barnyard grass species to the new herbicides, florpyrauxifen-benzyl and tripyrasulfone, was tested. Three gradient doses of the same herbicide led to a gradient trend for each barnyard grass (Figure 2a). Florpyrauxifen-benzyl, at 36 g a.i./ha, inhibited more than 90% of the fresh weight of two *E. crus-galli* var. *crus-galli* populations, 80–90% of the fresh weight of two *E. glabrescens* populations, and less than 80% of the fresh weight of two *E. crus-galli* var. *zelayensis* populations. Tripyrasulfone, at 270 g a.i./ha, inhibited more than 90% of the fresh weight of two *E. crus-galli* var. *crus-galli* populations, 70–80% of the fresh weight of two *E. glabrescens* populations, and less than 40% of the fresh weight of two *E. crus-galli* var. *zelayensis* populations. The decrease in the fresh weight of *E. crus-galli* var. *zelayensis* caused by the highest and second highest doses of the two herbicides was significantly lower than that of the other four populations of *E. crus-galli* var. *crus-galli* and *E. glabrescens* (*p* < 0.05) (Figure 2b).

### 2.3. Characteristics of Chloroplast Genomes

**Figure 3:**
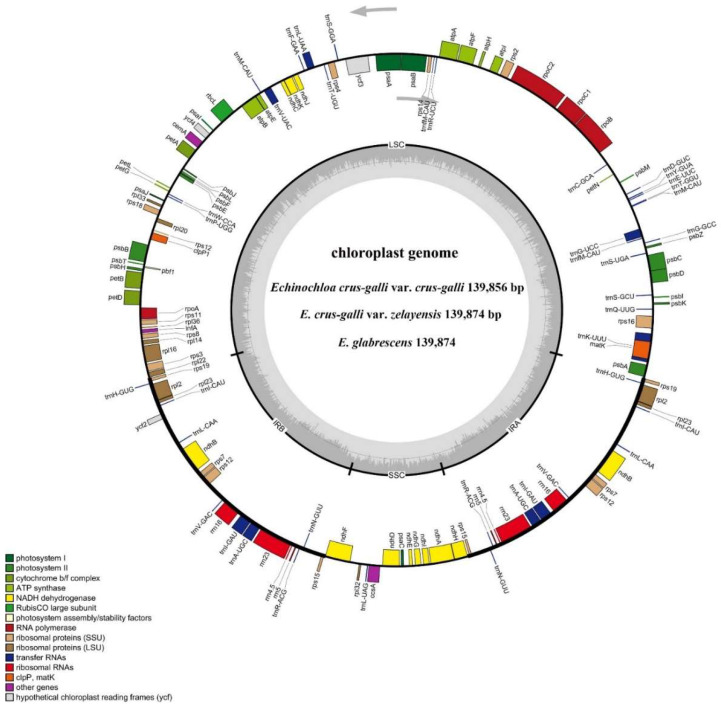
Assembly, size, and features of cp genomes of three *Echinochloa* spp. The genes outside the circle are transcribed in the counterclockwise direction, and the genes inside the circle are transcribed in the clockwise direction. Different colors in genes represent different functions. The dark gray area and light gray area of the inner circle represent the GC content to AT content of the genome, respectively.

The cp genome library of *E. crus-galli* var. *crus-galli*, *E. crus-galli* var. *zelayensis,* and *E. glabrescens* was constructed using the Illumina TruSeq Nano DNA Sample Prep Kit. After trimming low-quality fragments from the raw data, 51,498,928, 48,018,716, and 57,983,252 clean reads with 46.36%, 45.10%, and 45.51GC% were mapped to the complete genome of *E. crus-galli* var. *crus-galli*, *E. crus-galli* var. *zelayensis*, and *E. glabrescens*, respectively. The de novo assembly using NOVOPlasty v4.2 software (https://github.com/ndierckx/NOVOPlasty, accessed on 26 November 2021) resulted in a circular genome of 139,856, 139,874, and 139,874 bp in length (Figure 3). Raw reads were deposited in the NCBI GenBank database (accession number: PRJNA827798). All three complete cp genomes displayed the typical quadripartite structure of most angiosperms, including a large single-copy (LSC) and small single-copy (SSC) region, and a pair of inverted repeats (IRa and IRb). The lengths of the LSC and SSC regions, and IRs were 81,843, 12,517, and 22,748 bp in *E. crus-galli* var. *crus-galli*, and 81,890, 12,514, and 22,735 bp in *E. crus-galli* var. *zelayensis* and *E. glabrescens*; the intergenic region lengths were 79,775, 79,751, and 79,751 bp in *E. crus-galli* var. *crus-galli*, *E. crus-galli* var. *zelayensis*, and *E. glabrescens*, respectively. The cp genome of all three barnyard grass genes contained 132 genes, including 84 protein-coding genes (Table 2).

### 2.4. Chloroplast Genome Component

**Figure 4:**
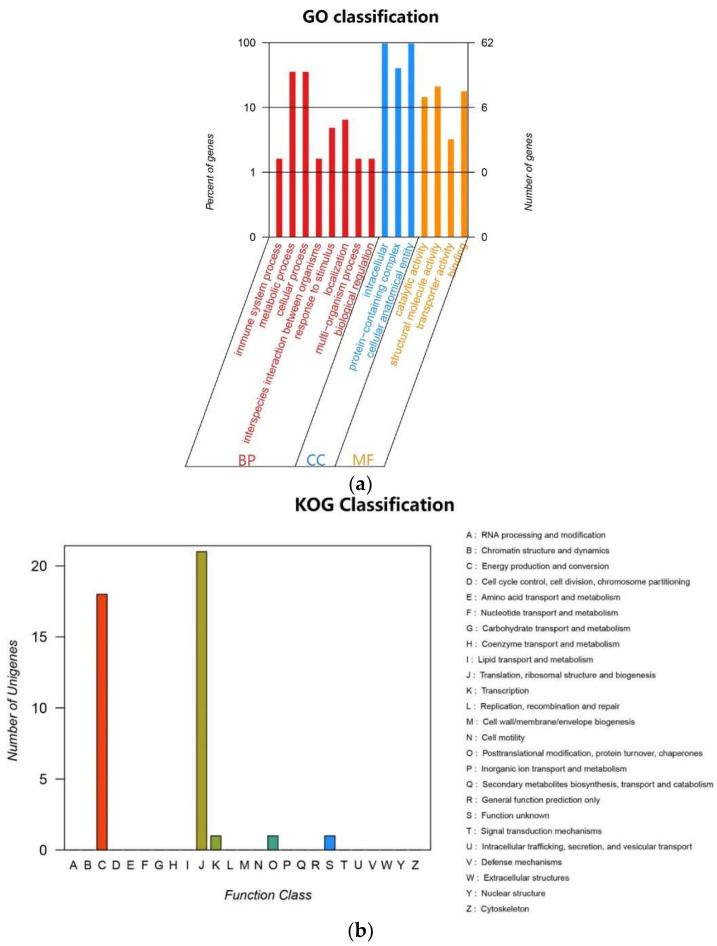
Classifications of gene functions of *E. crus-galli* var. *crus-galli*, *E. crus-galli* var. *zelayensis*, and *E. glabrescens*. (**a**) Percentages of genes matched to GO function classification. BP means biological process, CC means cellular component, and MF means molecular function. (**b**) Number of unigenes matched to KOG function classification.

The cp genome of *E. crus-galli* var. *crus-galli*, *E. crus-galli* var. *zelayensis,* and *E. glabrescens* contained 40 transfer RNA (tRNA) genes and eight ribosomal RNA (rrn) (Table 3). There were 67 protein-coding and 27 tRNA genes located within the LSC region, 12 protein-coding genes, 10 tRNA-coding genes, and four rRNA-coding genes located within IRb or IRa, and 11 protein-coding and one tRNA gene located within the SSC region (Figure 3). All 84 genes encoding proteins in the cp genome of these three barnyard grass species were functionally annotated in this study, mainly belonging to the photosynthesis and self-replication categories. The gene names, groups, and categories are listed in Table 4. Genes mainly belonged to biological processes in GO (Figure 4a) and were mainly involved in energy production and conversion, translocation, ribosomal structure and biogenesis, and transcription pathways in KEGG (Figure 4b). A total of 136, 139, and 139 simple sequence repeats (SSRs) were identified in *E. crus-galli* var. *crus-galli*, *E. crus-galli* var. *zelayensis,* and *E. glabrescens* cp genomes. There were five SSRs on IRa or IRb, 110 SSRs on the LSC, and 16 SSRs on the SSC in *E. crus-galli* var. *crus-galli*, and five SSRs on IRa or IRb, 113 on the LSC, and 16 on the SSC in *E. crus-galli* var. *zelayensis* or *E. glabrescens*. There were 62 long repeats (LRs) in *E. crus-galli* var. *crus-gall* and 68 LRs in *E. crus-galli* var. *zelayensis* and *E. glabrescens* (Table 5).

### 2.5. Single-Nucleotide Polymorphism Analysis

Single-nucleotide polymorphism analysis was performed to further explore the DNA sequence polymorphisms and differences caused by single-nucleotide variations in *E. crus-galli* var. *crus-galli*, *E. crus-galli* var. *zelayensis*, and *E. glabrescens*. The results indicate that 321 SNPs were detected in *E. crus-galli* var. *crus-galli*, representing 223 in intergenic spacer (IGS) regions, 98 in CDS regions (Supporting Table S1), and only one SNP IGS region of the *E. glabrescens* cp genome, compared to *E. crus-galli* var. *zelayensis* (Table 6). One SNP appeared in the stop codon, and 76 synonymous mutations and 21 non-synonymous mutations in *E. crus-galli* var. *crus-galli*. A total of 21 non-synonymous mutations were found in 14 coding genes, including *matK*, *psbC*, *rpoC1*, *rpoC2*, *atpF*, *atpE*, *rbcL*, *petA*, *petD*, *rpoA*, *rpl22*, *ndhF*, *ndhA*, and *ndhH* (Supporting Table S1). The non-synonymous to synonymous substitution (dN/dS) ratio was 0.28.

### 2.6. IR Expansion and Contraction

**Figure 5:**
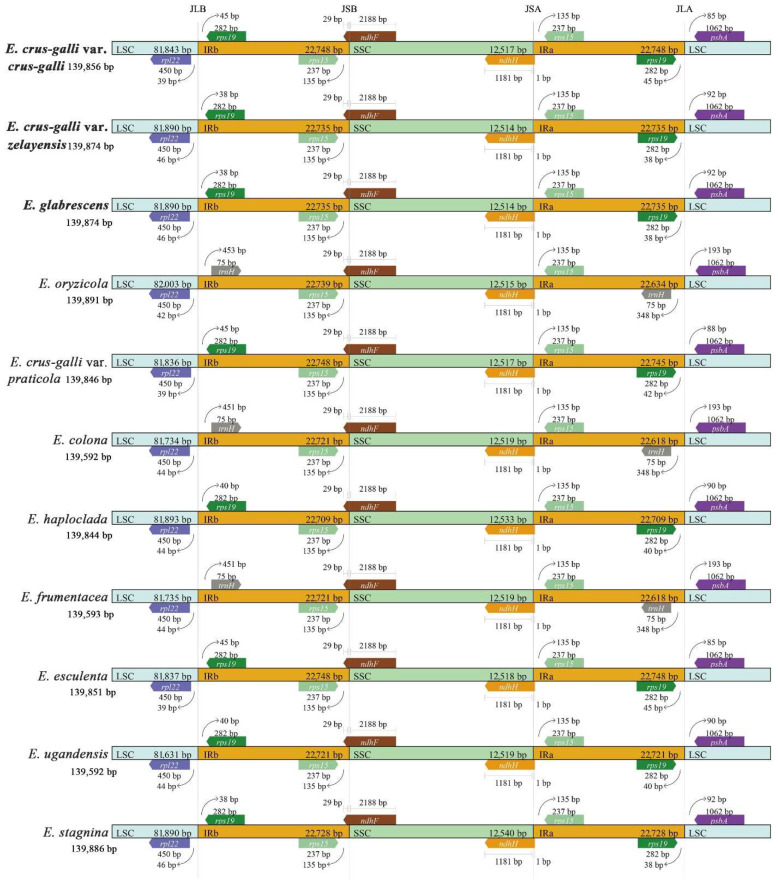
Comparison of LSC, IRb, SSC, and IRa border regions in ten species of *Echinochloa* spp. The species in bold font were sequenced in this study.

To further observe the potential contraction and expansion of the IR regions, the gene variations at the IR/SSC and IR/LSC boundary regions of ten sedges were compared (Figure 5). The *rps19*/*rpl22*, *rps15*/*ndhF*, *ndhH*/*rps15*, and *rps19*/*psbA* genes were located on the junctions of IRb/LSC, IRb/SSC, IRa/SSC, and IRa/LSC regions in *E. crus-galli* var. *crus-galli*, *E. crus-galli* var. *zelayensis*, and *E. glabrescens*. The junction genes at IRb/LSC and IRa/LSC of *E. colona*, *E. frumentacea*, and *E. oryzicola* differ from those of our three species, namely, *rpl22/trnH* and *trnH*/*psbA*. The length of the junction genes was consistent in *E. crus-galli* var. *crus-galli*, *E. crus-galli* var. *zelayensis*, and *E. glabrescens*. The *rpl22* gene located in the LSC region was 39 bp from the IRb region in *E. crus-galli* var. *crus-galli*, whereas the distance in *E. crus-galli* var. *zelayensis* and *E. glabrescens* was 46 bp long. The gene, *rps19*, located in the IRa region of *E. crus-galli* var. *crus-galli* was 45 bp from the LSC region, whereas the distance in *E. crus-galli* var. *zelayensis* and *E. glabrescens* was 38 bp long. The *psbA* gene, located in the LSC region of *E. crus-galli* var. *crus-galli,* was 85 bp from the IRa region, whereas the distance in *E. crus-galli* var. *zelayensis* and *E. glabrescens* was 92 bp long. The length and distance from the boundaries of junction genes located in IRb/SSC and IRa/SSC regions were consistent in eleven barnyard grass species.

### 2.7. Phylogenetic Analysis

**Figure 6:**
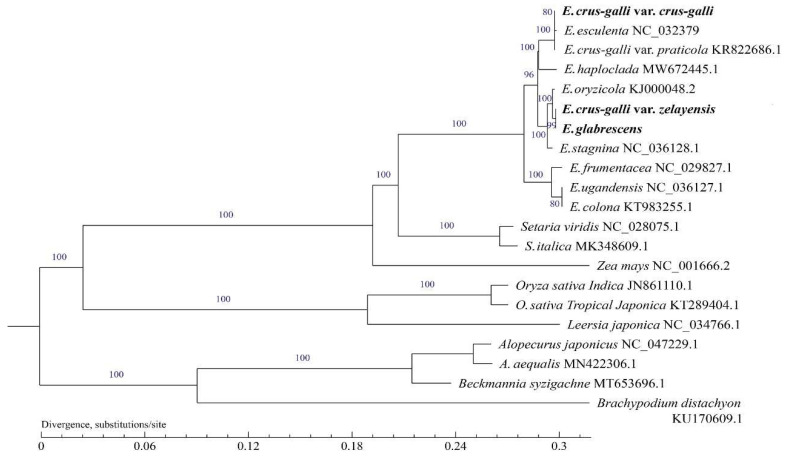
Phylogenetic tree for 20 species of Gramineae using maximum likelihood, based on alignments of complete chloroplast genomes. The numbers at the nodes indicate bootstrap values from 1000 replicates. If the bootstrap values are 100, this number was not shown on the nodes. The species in bold font were sequenced in this study.

Phylogenetic trees were generated using maximum likelihood (ML) and Bayesian inference (BI) analysis methods based on 21 complete cp genomes showing the same topology (Figure 6). *Echinochloa* spp. were clustered into a single clade. *E. crus-galli* var. *zelayensis* and *E. glabrescens* have the most recent common ancestor (MRCA) (BS = 99 for ML), which has an MRCA with *E. oryzicola* (BS = 100 for ML). The closest relative to the above three *Echinochloa* spp. is *E. stagnina* (BS = 100 for ML). The species close to the above four *Echinochloa* spp. is *E. crus-galli* var. *crus-galli* sequenced in the present study (BS = 98 for ML). Although *E. crus-galli* var. *crus-galli* and *E. esculenta* were found to be closely related, the BS value was only 80 for ML. Among all the different plant species we collected, the closest relationship with the genus *Echinochloa* was the genus *Setaria* (BS = 100 for ML), the second most closely related to *Zea mays* (BS = 100 for ML), and the third most closely related to the genus *Oryza* (BS = 100 for ML). *Alopecurus* spp., *Beckmannia syzigachne*, and *Brachypodium distachyum* were all on another branch of the phylogenetic tree.

## 3. Discussion

Studies have distinguished the species of barnyard grass weeds mainly according to their morphology after budding [11]. The awn length is significantly different in the seeds of different barnyard grass species. Although Ruiz-Santailla et al. found that the awn length of barnyard grass is related to the growth environment [12], the differences in awn length between some barnyard grass species are still very prominent. In the present study, the morphological differences in seeds were the main basis for identifying several barnyard grass species (Figure 1b). The seeds of *E. crus-galli* var. *crus-galli* awns are 1–2 cm long at the top, and *E. glabrescens* seeds are convex on both sides, bright leather, and heavier, which are important distinguishing features for the two barnyard grass species. However, the seeds of *E. crus-galli* var. *zelayensis* had no prominent identification characteristics. More importantly, it is difficult to identify barnyard grass at the seedling stage (Figure 1a), which is the key period for herbicide selection. Therefore, it is important to determine the differences among barnyard grass species using chloroplast genome sequencing.

Barnyard grass is one of the most troublesome weeds in paddy fields [3,4,7,8]. The genus has many species that are difficult to distinguish, and their genetic relationships are complex; however, related research is still not systematic. Previous studies have reported differences in the sensitivity of different barnyard grasses to one herbicide [9,10]. The herbicide sensitivity test in this study demonstrated the importance of identifying barnyard grass species. Barnyard grass has evolved resistance to many post-emergence herbicides in China [3,4,41,42,43]. Therefore, in this study, two new herbicides, florpyrauxifen-benzyl and tripyrasulfone, that have not been widely used in paddy fields in China but have the potential to control barnyard grass were selected to test the difference in tolerance to herbicides. *E. crus-galli* var. *crus-galli* and *E. glabrescens* were susceptible to two herbicides; therefore, tripyrasulfone, owing to its lower cost, can be selected for the management of these species. Meanwhile, the control effect of florpyrauxifen-benzyl on *E. crus-galli* var. *zelayensis* is significantly better than that of tripyrasulfone (<40%), so florpyrauxifen-benzyl should be selected in this case despite its higher price. Therefore, accurately identifying barnyard grass species is key to selecting suitable herbicides.

Since the complete chloroplast (cp) genome sequence of tobacco was first reported [44], many plant cp genome sequences have been determined [36,40,45,46]. Although the cp genomes of many barnyard grass species have been sequenced [47], those of *E. crus-galli* var. *zelayensis* and *E. glabrescens* have not yet been reported. In this study, the cp genome of *E. crus-galli* var. *crus-galli*, *E. crus-galli* var. *zelayensis,* and *E. glabrescens* were sequenced, which showed that the genomes of the three species were similar in size (Figure 3). The reported cp genomes of barnyard grass species are between 139,592 and 139,891 bp in size [47], indicating that the differentiation of cp genome size in the genus is not prominent. The cp genome of barnyard grass is relatively small compared with that of many terrestrial plants [27,40,45,46]. The typical tetrad structure of the chloroplast genome is conserved in plants [31,32,33], and, generally, there is little difference in the length of each tetrad of the same genus [27,40,46]. A tetrameric structure exists in the cp genomes of all three barnyard grass species sequenced in our study, and the length difference of each region was only within 3–47 bp. From the GC content perspective, the differences among the three barnyard grass species are only within 0.02% (Table 2). The parameters were consistent in *E. crus-galli* var. *zelayensis* and *E. glabrescens*, preliminarily implying that they have a relatively close genetic relationship.

The chloroplast genome is highly conserved in plants of the same genus [24,25,26]. The number of coding, tRNA, and rrn genes in the cp genomes of *E. crus-galli* var. *crus-galli*, *E. crus-galli* var. *zelayensis,* and *E. glabrescens* were completely consistent. Furthermore, their distribution in the four tetramerous structures was also consistent (Figure 3). Simultaneously, the annotated coding genes also maintained a high degree of consistency among the three barnyard grass species (Table 4). These results confirm the conservation of the cp genome among the three barnyard grass species. SSRs, also known as microsatellites, are widely distributed in plant cp genomes and are composed of one–six-nucleotide repeat units [48,49]. These repetitive structures exhibit diversity among cp genomes in the population and promote molecular recombination [50]. SSRs are an important molecular genetic marker now widely used in population genetics and plant genotyping [51,52,53,54]. This study showed three more SSRs in the cp genome of *E. crus-galli* var. *zelayensis* and *E. glabrescens* than in *E. crus-galli* var. *crus-galli*, and one less in the coding region (Table 5). Differential SSRs can be used as a specific molecular marker for this species. LRs usually occupy a large proportion of the genome, which is also a special and repeated DNA sequence [55]. Repeat fragments have an important molecular significance in the study of plant evolution [56]. The number and distribution of LRs in the cp genome of *E. crus-galli* var. *zelayensis* and *E. glabrescens* were consistent, three times higher than that of *E. crus-galli* var. *crus-galli* (Table 5). The repeats identified in this study are of great significance for the species identification, genetic diversity, and population structure of the genus *Echinochloa*. The cp genomes of *E. crus-galli* var. *zelayensis* and *E. glabrescens* were more similar. Concurrently, there were limited differences between the two barnyard grass species and *E. crus-galli* var. *crus-galli*.

Recently, SNPs have become a key tool and measurement indicator in evolution and classification research because many samples can be screened by cheap high-throughput technology [57]. They can display the exact nature and location of allele variation, which is widely used as a direct marker [58]. Researchers have successfully distinguished white, black, and red spruces using SNPs as molecular markers [58]. In the present study, compared with the cp genome of *E. crus-galli* var. *zelayensis*, 97 SNPs were detected in the *E. crus-galli* var. *crus-gall* and the number of non-synonymous SNPs is lower than that of synonymous SNPs, indicating no strong diversification between the two barnyard grass species [59]. These SNPs can be used as important differential nucleotide databases to distinguish barnyard grass species. Simultaneously, this study showed a close genetic relationship between *E. crus-galli* var. *zelayensis* and *E. glabrescens* because only one mutation was detected in the cp genome between them. SNPs usually occur more frequently in variable and less conserved genes [58], which was also confirmed by our study. McDonald et al. proposed that repeat-induced recurrence repair is the mechanism underlying SNP induction [60]. The mechanism of *Echinochloa* differentiation and SNP production requires further research.

The expansion and contraction of the chloroplast genome mainly occur at the junction of IR/SC [61], a very common biological phenomenon in plants [24]. Although highly conserved, IR expansion and contraction are important driving forces of genome evolution because they are directly related to variations in cp genome size and rearrangement [62,63,64,65]. This phenomenon has been repeatedly observed in many plants [27,36,40]. This study showed that, compared with *E. crus-galli* var. *zelayensis*, *E. glabrescens* has no expansion or contraction of the IR, whereas *E. crus-galli* var. *crus-galli* showed minimal change. Furthermore, there was no difference among the three barnyard grass species in the adjacent genes of junctions, genes across regions, and the length of these genes. Differences in *E. crus-galli* var. *crus-galli* were mainly caused by the distance between the boundary gene and the boundary, but this change was only 7 bp (Figure 5). Our IR expansion and contraction results fully demonstrate the high conservatism of barnyard grass. Genes, gene length, and distance from the boundary at the junction of IRs and SSCs were completely consistent between the 10 barnyard grass species (Figure 5). The main difference was the boundary gene between IRs and LSC regions, which is *trnH* in *E. colona*, *E. frumentacea*, and *E. oryzicola*, but *rps19* in other barnyard grass species (Figure 5), inconsistent with the genetic relationship among barnyard grass species previously reported [13,14,15,16,19,66]. Our results provide a novel idea for studying the genetic relationships of the genus *Echinochloa*, and the differentiation mechanism needs to be further explored.

The cp genome is essential for plant phylogeny and species identification [67,68,69]. CP genome data can also establish organelle-based “barcodes” for some species, which is valuable for establishing species definition because it is then used to reveal phylogenetic relationships [70]. The main method consists in constructing a phylogenetic tree based on the cp genome. With the continuous development of cp genome information and technological improvements, the genetic and evolutionary relationships of many plants have been successfully clarified [27,46,71]. However, the genetic evolution of the genus *Echinochloa* from the perspective of the cp genome has not yet been reported. In this study, we collected all reported cp genomes of barnyard grass and some cp genomes of representative grasses from the NCBI to conduct phylogenetic analysis. The cp genome data of *E. crus-galli* var. *zelayensis* and *E. glabrescens* were measured and published for the first time (accession number: PRJNA827798). According to the results of genetic relationships, the 10 species of barnyard grass in this study can be divided into four groups. The first group comprises *E. oryzicola*, *E. crus-galli* var. *zelayensis*, *E. glabrescens*, and *E. stagnina*; the second group includes *E. crus-galli* var. *crus-galli*, and *E. esculenta*; the third group contains *E. haploclada* alone; and the fourth group consists of *E. ugandensis*, *E. colona*, and *E. frumentacea*. The genus *Echinochloa* is closely related to the genus *Setaria* (Figure 6), which is why many genes of the genus *Echinochloa* weeds can be matched to those of the genus *Setaria* [72,73]. Although analysis of the complete cp genome may not be sufficient to fully solve all phylogenetic relationships, it can still provide a feasible way to clarify species relationships [68,74,75].

## 4. Materials and Methods

### 4.1. Plant Materials

Two *E. crus-galli* var. *zelayensis* populations were provided by the Herbicide Research Laboratory of Nanjing Agricultural University, China [3]. In addition, two *E. crus-galli* var. *crus-galli* and two *E. glabrescens* populations were collected from paddy fields in the Yangtze River Delta, China, in 2020. All six populations were tested for whole-plant bioassays and seed morphology. In addition, one population of each barnyard grass species was subjected to chloroplast (cp) genome sequencing.

### 4.2. Measurement of Awn Length and Seed Weight

Barnyard grass seeds were dried to a constant weight under the sun before the test. Thirty seeds of each barnyard grass species were randomly selected, and the awn length of the seeds was measured. A total of 1000 seeds of each species of barnyard grass were randomly selected as a group for weight determination, and six groups were used as replicates. The experimental groups were randomly arranged. The data were subjected to ANOVA. To compare the differences in awn length and seed weight among the three barnyard grass species, Duncan’s multiple range test (*p* < 0.05) was used. ANOVA was performed using SPSS version 20 (SPSS, Chicago, IL, USA).

### 4.3. Whole-Plant Bioassay to Determine Sensitivity to New Herbicides

The stems and leaves of six barnyard grass populations belonging to three species were sprayed with florpyrauxifen-benzyl (Corteva Agriscience, Wilmington, DE, USA) or tripyrasulfone (KingAgroot, Qingdao, Shandong Province, China) when the plants reached the 3–4-leaf stage using a 3WP-2000 walking-type spraying system (Nanjing, China). The spraying system was equipped with a 390 mL/min flow nozzle with a pressure of 3.0 kg/cm^2^ at the time of spraying. When spraying herbicides, whole plants grown in pots were placed in the spraying system, and 30 mL of the diluted herbicide solution was sprayed onto the plants at a forward speed of 291 mm/s through the nozzle to ensure that the droplets of quinclorac solution that fell on the plants were small and uniform enough and that the final doses were 9, 18, and 36 g a.i. ha^−1^ for florpyrauxifen-benzyl and 67.5, 135, and 270 g a.i. ha^−1^ for tripyrasulfone. Each experimental treatment contained four biological replicates, and the experiment was conducted twice.

All studies were conducted using the inhibition rate (IR) of fresh weight, which is based on the fresh weight of CK. The experimental groups were randomly arranged. The data were subjected to ANOVA. To compare the differences in the percentage of inhibition rate among the 18 groups, Duncan’s multiple range test (*p* < 0.05) was used. ANOVA was performed using SPSS version 20 (SPSS, Chicago, IL, USA).

IR = (W_CK_ − W_T_)_/_W_CK_ × 100%

where W_CK_ represents the fresh weight of the plants in the untreated group and W_T_ represents the fresh weight of the plants in the treatment group.

### 4.4. DNA Extraction and Sequencing

Fresh leaves and stems of total genomic DNA were extracted using a modified cetyltrimethylammonium bromide (CTAB) method and applied to 500 bp paired-end library construction using the NEBNext Ultra DNA Library Prep Kit for Illumina sequencing. Sequencing was performed on an Illumina NovaSeq 6000 platform (BIOZERON Co., Ltd., Shanghai, China). Raw data from three barnyard grass species were generated with 150 bp paired-end read lengths.

### 4.5. DNA Sequencing and Genome Assembly

De novo assembly with NOVOPlasty, referencing the cp genome of closely related species, produced two optional circular contigs of the cp genome. One of them, with higher homology cpDNA, was selected as the candidate cp genome. Several potential chloroplast reads were extracted from the pool of Illumina reads using BLAST searches against the cp genomes of related species *E. stagnina voucher* K: RCH49 chloroplast (Accession Number: MF563381) and the NOVOPlasty results. Illumina chloroplast reads were obtained to perform cp genome de novo assembly using the SPAdes-3.13.0 package. The NOVOPlasty assembly contig was optimized by the scaffolds from the SPAdes-3.13.0 result and aligned with the original clean Illumina reads using the BWA, and the base correction was performed with Pilon v1.22. Finally, the assembled sequence was reordered and oriented according to the reference cp genome to generate the final assembled chloroplast genomic sequence.

### 4.6. Genome Component Analysis

Genes encoding proteins, tRNAs, and rRNAs in the chloroplast genome of *E. crus-galli* var. *crus-galli*, *E. crus-galli* var. *zelayensis*, and *E. glabrescens* were predicted using the GeSeq (https://chlorobox.mpimp-golm.mpg.de/geseq.html/, accessed on 26 November 2021). The specific parameters were set as follows: protein search identity: 60; rRNA, tRNA, DNA search identity: 35; 3rd party tRNA annotators: tRNAscan-SE v2.0.7. High-accuracy gene bundles were obtained by removing the redundancy of predicted initial genes, followed by manual correction of the head, tail, and exon/intron boundaries of the genes. Finally, the base composition of the chloroplast genome; the gene distribution of each interval, including the large single-copy (LSC) regiom, small single-copy (SSC) region, and inverted repeats (IRs); and the classification of each functional gene was counted and summarized.

### 4.7. Gene Function Annotation and Classification Analysis

The protein sequences of chloroplast genes were compared with known protein databases using BLASTP (evalue < 1 × 10^−5^). Since there may be more than one alignment result for each sequence, to ensure its biological significance, only one optimal alignment result was reserved as the database alignment information of the gene. These databases included NR (http://www.ncbi.nlm.nih.gov/, accessed on 26 November 2021), Swiss-Prot (http://www.ebi.ac.uk/uniprot, accessed on 26 November 2021), eggNOG (http://eggnogdb.embl.de/, accessed on 26 November 2021), KEGG (http://www.genome.jp/kegg/, accessed on 26 November 2021), and GO (http://geneontology.org/, accessed on 26 November 2021). The amino acid sequences of *C. difformis* and *C. iria* were aligned with the NR, Swiss-Prot, eggNOG, KEGG, and GO databases to obtain functional annotation information for the coding genes.

### 4.8. Contraction and Expansion Analysis of Inverted Repeat (IR) Regions

In this part, in addition to the three newly sequenced cp genomes of barnyard grass, eight other barnyard grass species and an additional 10 Gramineae plant cp genomes were downloaded from NCBI to resolve the IR analysis. The four quadripartite structures of each chloroplast (LSC, SSC, and two IR repeat regions) were compared, and changes in the copy number of related genes caused by contraction and expansion of the IR or pseudogenes resulting in boundary regions were analyzed. Genes that crossed the boundary or genes closest to the boundary were obtained. The function, length, and distance from the boundaries of these genes were analyzed.

### 4.9. Phylogenetic Analysis

In this part, in addition to the three newly sequenced cp genomes of barnyard grass, eight other barnyard grass species and an additional 10 Gramineae plants were downloaded from NCBI to resolve a chloroplast phylogenetic tree. The sequences were aligned using ClustalW (v2.0.12) with the default settings. The DNA substitution model was assessed using the Akaike information criterion (AIC) method [76]. The phylogenetic tree was constructed by the maximum likelihood (ML) method using PhyML v3.0 (htp://ww.atgc-montpeller.fr/phyml/, accessed on 19 October 2022), and the bootstrap was 1000 [77,78]. Bayesian inference (BI) was also used based on the method described by Wu et al. [79], using MrBayes v3.1.2.

## 5. Conclusions

The cp genomes of *E. crus-galli* var. *zelayensis* and *E. glabrescens* were first sequenced, revealing a close relationship in our study. Although *E. crus-galli* var. *zelayensis*, *E. glabrescens*, and *E. crus-galli* var. *crus-galli* were very similar in morphology at the seedling stage, *E. crus-galli* var. *crus-galli* showed some differences in size, components, gene annotation, repeats, and IR expansion and contraction of the cp genome. The SNP results further revealed a close relationship between *E. crus-galli* var. *zelayensis* and *E. glabrescens*. The detected SNPs can be used to conveniently identify the three barnyard grass species. Furthermore, IR expansion and contraction and the phylogenetic tree illustrated differences in the evolutionary directions of the genus *Echinochloa*, which is the molecular basis of biodiversity. The results also provide important biological information for the identification and evolution of the genus *Echinochloa*. However, the mechanisms that cause the substantial differentiation of the genus *Echinochloa* and the difference of herbicide sensitivity are still unclear and need further study.

## Figures and Tables

**Table 1:** Differences in seeds of three *Chinchaga* spp.

*Echinochloa* spp.	Length of Awn (cm)	Dry Weight Per 1000 Seeds (g)
*E. crusgalli* var. *crusgalli*	1.63 ± 0.05 a	2.806 ± 0.011 b
*E. crusgalli* var. *zelayensis*	0.00 ± 0.00 b	2.139 ± 0.016 c
*E. glabrescens*	0.00 ± 0.00 b	3.006 ± 0.032 a

“a–c” are the significance levels at different lengths of awn or dry weight per 1000 seeds of each barnyard grass species.

**Table 2:** Summary of chloroplast genome features in three barnyard grass biotypes.

Genome Features	*E. crus-galli* var. *crus-galli*	*E. crus-galli* var. *zelayensis*	*E. glabrescens*
Genome size (bp)	139,856	139,874	139,874
LSC length (bp)	81,843	81,890	81,890
SSC length (bp)	12,517	12,514	12,514
IR length (bp)	22,748	22,735	22,735
Intergenic region length (bp)	79,775	79,751	79,751
Overall GC content (%)	38.63	38.63	38.63
GC content of LSC (%)	36.47	36.46	36.46
GC content of SSC (%)	33.21	33.22	33.22
GC content of IRs (%)	44.01	44.03	44.02
Total genes	132	132	132
Number of protein-coding genes	84	84	84

**Table 3:** Non-coding RNA statistics.

*Echinochloa* spp.	Type	ncRNA Number	Total Length (bp)	Average Length (bp)	Length/ Genome (%)
*E. crus-galli* var. *crus-galli*	tRNA	40	2972 *	74	2.13
	rrn23	2	**5778**	**2889**	**4.13**
	rrn4.5	2	190	95	0.14
	rrn16	2	2982	1491	2.13
	rrn5	2	242	121	0.17
*E. crus-galli* var. *zelayensis*	tRNA	40	**2976**	74	2.13
	rrn23	2	**5579**	**2789**	**3.99**
	rrn4.5	2	190	95	0.14
	rrn16	2	2982	1491	2.13
	rrn5	2	242	121	0.17
*E. glabrescens*	tRNA	40	**2976**	74	2.13
	rrn23	2	**5380**	**2690**	**3.85**
	rrn4.5	2	190	95	0.14
	rrn16	2	2982	1491	2.13
	rrn5	2	242	121	0.17

* The bold number indicates that the data are different among the three barnyard grass species.

**Table 4:** Genes encoded by three species of *Echinochloa* chloroplast genome.

Category	Groups	Genes
Photosynthesis	Subunits_of_photosystem_I	*psaA*, *psaB*, *psaC*, *psaI*, and *psaJ*
	Subunits_of_photosystem_II	*pbsN*, *psbA*, *psbB*, *psbC*, *psbD*, *psbE*, *psbF*, *psbH*, *psbI*, *psbJ*, *psbK*, *psbL*, *psbM*, *psbT*, and *psbZ*
	Subunits_of_NADH_dehydrogenase	*ndhA*, *ndhB*, *ndhB*, *ndhC*, *ndhD*, *ndhE*, *ndhF*, *ndhG*, *ndhH*, *ndhI*, *ndhJ*, and *ndhK*
	Subunits_of_cytochrome_b/f_complex	*petA*, *petB*, *petD*, *petG*, *petL*, and *petN*
	Subunits_of_ATP_synthase	*atpA*, *atpB*, *atpE*, *atpF*, *atpH*, and *atpI*
	Large_subunit_of_Rubisco	*rbcL*
Self-replication	Large_subunits_of_ribosome	*rpl14*, *rpl16*, *rpl2*, *rpl2*, *rpl20*, *rpl22*, *rpl23*, *rpl23*, *rpl32*, *rpl33*, and *rpl36*
	Small_subunits_of_ribosome	*rps11*, *rps12*, *rps12*, *rps14*, *rps15*, *rps15*, *rps16*, *rps18*, *rps19*, *rps19*, *rps2*, *rps3*, *rps4*, *rps7*, *rps7*, and *rps8*
	DNA-dependent_RNA_polymerase	*rpoA*, *rpoB*, *rpoC1*, and *rpoC2*
	Ribosomal_RNAs	*8 rRNA*
	Transfer_RNAs	*40 tRNAs*
Other genes	Maturase	*matK*
	Protease	*clpP1*
	Envelope_membrane_protein	*cemA*
	Acetyl-CoA_carboxylase	
	C-type_cytochrome_synthesis_gene	*ccsA*
	Translation_initiation_factor	*infA*
	protochlorophillide_reductase_subunit	
Genes of unknown function	Proteins_of_unknown_function	*ycf2*, *ycf3*, and *ycf4*

**Table 5:** SSRs and LRs in three barnyard grass biotypes.

Types of Repeats		*E. crus-galli* var. *crus-galli*	*E. crus-galli* var. *zelayensis*	*E. glabrescens*
SSR Region Distribution	Genome	136	139	139
	Coding	43	42	42
	IRa	5	5	5
	IRb	5	5	5
	LSC	110	113	113
	SSC	16	16	16
LR Hamming Distance	0	8	9	9
	1	0	2	2
	2	21	21	21
	3	33	36	36

**Table 6:** Single-Nucleotide Polymorphism (SNP) in *Echinochloa crus-galli* var. *crus-galli* and *E. glabrescens* compared to *E. crus-galli* var. *crus-galli*.

Barnyard Grass Species	Start	Stop	Synonymous	Non-Synonymous	CDS	Intergenic	Total_SNP
*E. crus-galli* var. *crus-galli*	0	1	76	21	98	223	321
*E. glabrescens*	0	0	0	0	0	1	1

## Data Availability

Raw reads of cp genomes of *E. crus-galli* var. *crus-galli*, *E. crus-galli* var. *zelayensis*, and *E. glabrescens* were deposited in the NCBI GenBank database (accession number: PRJNA827798).

## Supplementary Materials

The following supporting information can be downloaded at: https://www.mdpi.com/article/10.3390/ijms232213864/s1.
